# Effect of intramedullary nail length on the biomechanical performance of internal fixation for subtrochanteric femoral fractures

**DOI:** 10.1186/s12891-026-09991-8

**Published:** 2026-05-22

**Authors:** Haitao Lu, Fang Chen, Qinghua Cheng, Zhanpo Wu, Zhi Xu, Changzheng Guo, Xiaolei Sheng

**Affiliations:** 1 Department of Orthopedics, Zhongda Hospital Lishui Branch, Southeast University, No.86, Chongwen Road, Yongyang Town, Lishui District, Nanjing, Jiangsu 211200 China; 2 Department of Orthopedics, Zhangjiagang Fifth People’s Hospital, Zhangjiagang, Jiangsu 215600 China; 3 Department of Orthopaedic Surgery, Zhangjiagang Hospital Affiliated to Soochow University, No.68, Jiyang West Road, Zhangjiagang, Jiangsu 215600 China

**Keywords:** Subtrochanteric femoral fracture, Intramedullary nail length, Reconstruction plate, Finite element analysis, Biomechanics

## Abstract

**Objective:**

To compare the biomechanical performance of different lengths of intramedullary nails combined with reconstruction plates for fixation of Seinsheimer type IV subtrochanteric femoral fractures using finite element analysis, with additional evaluation of osteoporotic bone conditions.

**Methods:**

A three-dimensional finite element model of Seinsheimer type IV subtrochanteric femoral fracture was constructed from CT data of a 51-year-old male volunteer. Three PFNA intramedullary nail lengths combined with a reconstruction plate were modelled: long-nail (320 mm), medium-nail (240 mm), and short-nail (160 mm) combinations. Each construct was analysed under axial (2,100 N), bending (175 N), and torsional (15 N·m) loading conditions in both healthy and osteoporotic bone subgroups (elastic modulus reduced to 60% of normal). Overall displacement, femoral stress, implant stress, and load-sharing ratio were evaluated.

**Results:**

Under axial loading, the medium-nail combination exhibited the smallest maximum femoral displacement (10.7 mm healthy; 13.3 mm osteoporosis), while the short-nail combination showed the largest (13.2 mm; 19.7 mm) and the greatest sensitivity to bone quality. The short-nail combination generated the highest femoral stress under axial loading (325.4 MPa, 32.1% above the long-nail combination) and the highest nail stress under bending (199.4 MPa); the medium-nail combination produced notably high nail stress under torsion (300.7 MPa). The short-nail combination also exhibited substantially higher reconstruction plate stress under axial loading (804.5 MPa) relative to the long-nail (654.9 MPa) and medium-nail (578.8 MPa) combinations. Load-sharing analysis showed that the intramedullary nail bore the largest load fraction across all constructs (~ 47%–63%), and the long-nail combination demonstrated the most substantial load redistribution under osteoporotic conditions.

**Conclusion:**

The medium-nail combination may offer superior displacement control under axial loading, while the short-nail combination was associated with higher stress levels across multiple loading modes and greater biomechanical vulnerability in osteoporotic bone. Intramedullary nail length should be selected individually, accounting for fracture type, bone quality, and anticipated loading demands.

## Introduction

Subtrochanteric femoral fractures are a common type of hip fracture. Due to the high mechanical stress in this region and its predominantly cortical bone composition, these fractures are difficult to heal and challenging to treat [1, 2]. Intramedullary nail fixation has become the preferred treatment for subtrochanteric femoral fractures owing to its advantages of a shorter moment arm and reduced surgical trauma [3]. However, for comminuted fractures such as Seinsheimer type IV, the integrity of both the medial and lateral walls is compromised, making it difficult to maintain fracture stability with intramedullary nailing alone. Postoperative complications such as implant failure and fracture nonunion are therefore common [4, 5].

**Fig. 1:**
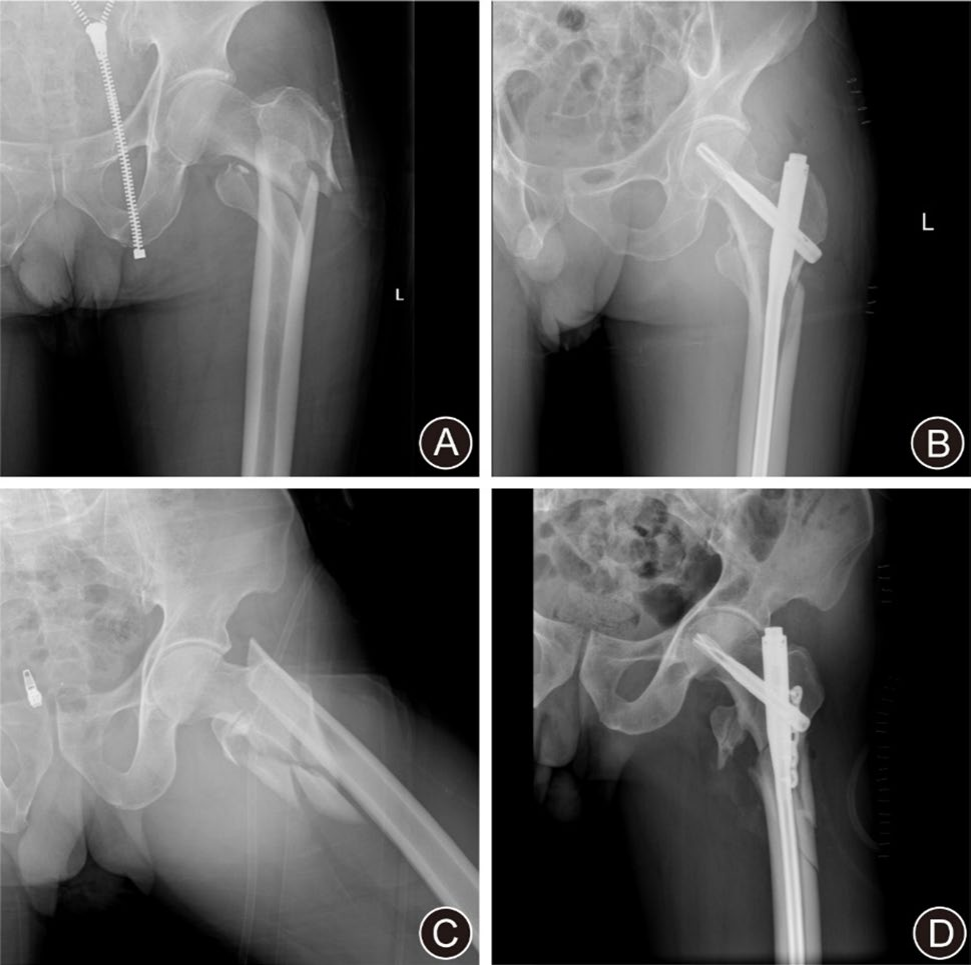
Representative cases. A 72-year-old male patient presented with left hip pain and limited range of motion after a fall. Figure **A** shows the preoperative anteroposterior radiograph demonstrating a left subtrochanteric femoral fracture (Seinsheimer type IV). Figure **B** shows the postoperative anteroposterior radiograph revealing satisfactory positioning of the intramedullary nail fixation, with the cephalomedullary screw centrally placed within the femoral head and neck, adequate restoration of the femoral neck-shaft angle, and the lesser trochanter fragment left unreduced and unfixed. A 57-year-old male patient presented with left hip pain and restricted range of motion following a fall injury. Figure **C** shows the preoperative anteroposterior radiograph demonstrating a left subtrochanteric femoral fracture (Seinsheimer type IV). Figure **D** shows the postoperative anteroposterior radiograph revealing satisfactory fracture reduction with well-positioned internal fixation implants

Recent literature has highlighted the critical importance of lateral wall integrity for the stability of internal fixation in subtrochanteric femoral fractures [6]. When the lateral wall is deficient or comminuted, the intramedullary nail loses its effective point of support, predisposing the fracture site to instability and fixation failure. The authors’ research group has firsthand clinical experience with this issue: in an early case of Seinsheimer type IV fracture (Fig. 1A–B), insufficient appreciation of the importance of lateral wall fixation led to the use of intramedullary nailing alone, with the free fracture fragment left inadequately managed. In contrast, a more recent case of Seinsheimer type IIA fracture (Fig. 1C–D), managed with a thorough understanding of lateral wall fixation, was treated intraoperatively with intramedullary nailing combined with a reconstruction plate to reinforce the lateral wall, achieving satisfactory reduction and fixation. This clinical experience suggests that for the more complex Seinsheimer type IV subtrochanteric femoral fracture, a “hybrid” fixation strategy combining an intramedullary nail with a reconstruction plate may be more appropriate [7, 8]. Nevertheless, there is currently no consensus on the optimal nail length when using this combined fixation approach. Some investigators have argued that longer intramedullary nails provide superior rotational stability and more uniform stress distribution [9], while others have proposed that shorter nails offer advantages in terms of reduced operative time, decreased intraoperative blood loss, and less disruption of the fracture-site vascularity [10]. The choice of nail length not only affects the complexity of the surgical procedure but also directly influences the biomechanical performance of the internal fixation construct [11].

Finite element analysis, as a well-established computational simulation technique, has been widely applied to the optimization of orthopedic internal fixation devices [12, 13]. Drawing on reflections from clinical practice, this study aimed to construct a finite element model of Seinsheimer type IV subtrochanteric femoral fracture using three-dimensional finite element analysis, and to compare the biomechanical performance of long, medium, and short intramedullary nails combined with a reconstruction plate under both healthy and osteoporotic bone conditions. The primary focus was on analyzing overall displacement, stress distribution under different loading conditions, with the goal of providing a theoretical basis for the clinical selection of appropriate intramedullary nail length.

## Materials and methods

### Subjects

One 51-year-old male volunteer was recruited from the orthopedic outpatient clinic. His height was 172 cm and body weight was 70 kg (body mass index: 23.6 kg/m²). He had no prior history of serious chronic underlying diseases or hip surgery. The left femur was assessed using a 64-slice spiral CT scanner (Siemens, Germany) with the patient in the supine position. The scan range extended from the proximal to the distal femur, ensuring complete coverage of the fracture zone and adjacent joint structures. Scanning parameters were as follows: tube voltage 120 kV, tube current 200 mA, slice thickness 1.0 mm, slice interval 0.5 mm, acquisition matrix 512 × 512, and pixel size 0.68 mm × 0.68 mm. The resulting two-dimensional tomographic images were saved in DICOM format. The volunteer was fully informed of the study purpose and procedures and participated on a voluntary basis with informed consent. All methods employed in this study were conducted in accordance with relevant regulations and guidelines. All protocols were approved by the Ethics Committee of Zhangjiagang Hospital Affiliated to Soochow University (ZJGYYLL-LW-2026-03-004).

### Finite element modeling methods

#### Construction of the subtrochanteric femoral fracture model

The acquired DICOM data were imported into Mimics 19.0 software (Materialise, Belgium) for threshold-based segmentation of the femur. A bone gray-value threshold of 226–3071 HU was selected to effectively separate soft tissue from bone tissue. Through a series of image processing steps including region growing, filling, and erasure, a three-dimensional model of the femur was extracted and saved in STL format. To further improve model accuracy, mesh smoothing and denoising were performed in 3-matic 11.0 software (Materialise, Belgium), yielding a three-dimensional femoral model with a smooth surface and well-defined details. In accordance with the Seinsheimer classification criteria, and in order to simulate a Seinsheimer type IV subtrochanteric femoral fracture with medial and lateral wall instability similar to the case shown in Fig. 1, the fracture line was positioned below the lesser trochanter. A 3 mm bone defect gap was created at the medial wall fracture site, and a wedge-shaped free fracture fragment was fashioned at the lateral wall to replicate the clinical presentation of a comminuted fracture. The model was then exported as an IGS file for subsequent analysis.

#### Construction of the three internal fixation models

**Fig. 2:**
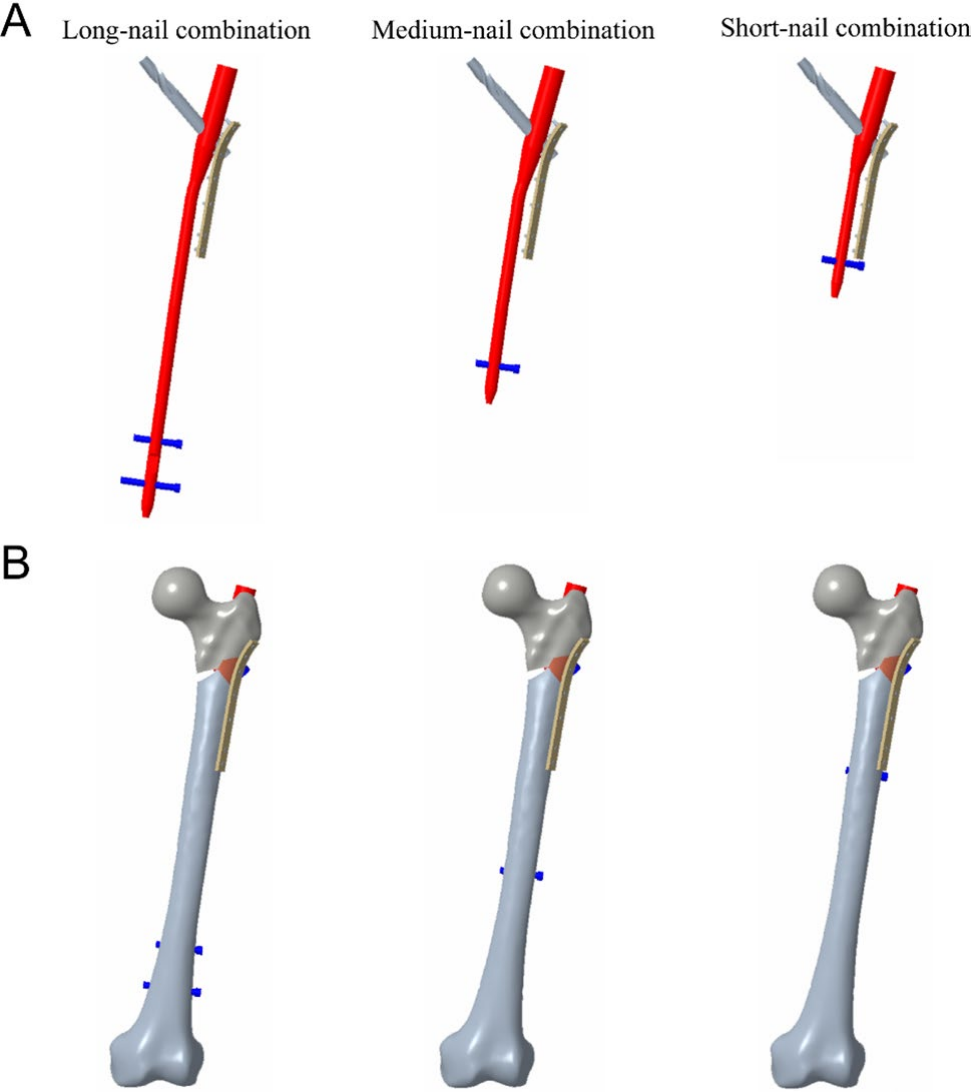
Finite element models of subtrochanteric femoral fracture fixation with three different lengths of intramedullary nails combined with reconstruction plates. **A** illustrates the three internal fixation construct models; (**B**) illustrates the three femur–implant assembly models

In this study, three geometric models of internal fixation using intramedullary nails of different lengths combined with a reconstruction plate were constructed, designated as the long-nail combination model, the medium-nail combination model, and the short-nail combination model (Fig. 2). Specifically, the long-nail combination model employed a long PFNA intramedullary nail (main nail length: 320 mm) combined with a reconstruction plate. The intramedullary nail was inserted via the tip of the greater trochanter, the helical blade was placed into the femoral head and neck, and the distal locking screw was fixed at the distal femoral shaft. The reconstruction plate was positioned on the lateral aspect of the femur to work in concert with the intramedullary nail to stabilize the fracture site. The medium-nail combination model employed a medium-length PFNA intramedullary nail (main nail length: 240 mm) combined with a reconstruction plate. The proximal fixation method was identical to that of the long-nail combination, while the distal locking screw was positioned at the mid-shaft of the femur. The reconstruction plate was placed laterally to provide supplementary fixation support. The short-nail combination model employed a short PFNA intramedullary nail (main nail length: 160 mm) combined with a reconstruction plate. The distal locking screw was positioned just below the fracture line, and the reconstruction plate assumed a greater proportion of load transmission in this configuration. Three-dimensional models of the internal fixation devices were constructed with reference to actual product specifications using Creo 6.0 software (PTC, USA). The helical blade, locking screws, reconstruction plate, and accompanying screws were all designed in accordance with the dimensions and positions used in clinical practice. The internal fixation models were then assembled with the femoral fracture model to form the femur–implant assembly models (Fig. 2B). Across the three models, nail length was the only intentionally varied parameter. All proximal implant features were held constant, including the insertion point (tip of the greater trochanter), helical blade dimensions, and its placement within the femoral head and neck, as well as the geometry and lateral positioning of the reconstruction plate. The distal locking screw position differed across models as a direct and clinically inherent consequence of nail length variation: at the distal femoral shaft in the long-nail model, at the mid-shaft in the medium-nail model, and just below the fracture line in the short-nail model. This co-variation reflects actual surgical practice and cannot be meaningfully separated from nail length selection.

#### Mesh generation and material property assignment

**Fig. 3:**
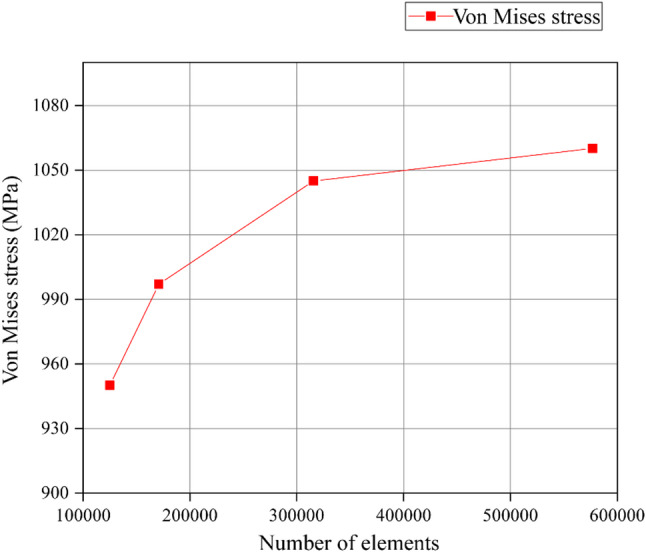
Grid sensitivity analysis

The three subtrochanteric femoral fracture internal fixation models developed through the above process were imported into HyperMesh 14.0 software (Altair, USA) as Parasolid assembly files. Following geometric cleanup, all solid components were meshed using four-node linear tetrahedral (C3D4) elements. Mesh convergence testing was performed using the medium-nail combination model, with multiple finite element models generated at different mesh resolutions: Mesh 1 (element count: 125,000; size: 1.5 mm), Mesh 2 (element count: 171,000; size: 1.2–1.5 mm), Mesh 3 (element count: 315,795; size: 1.0–1.2 mm), and Mesh 4 (element count: 577,000; size: 0.8–1.0 mm). The same load was applied to models of each mesh density, and changes in the maximum stress values were comparatively analyzed. Mesh convergence was considered achieved when the variation in stress values following further mesh refinement fell within 5% (Fig. 3) [14]. An adaptive mesh size of 1.0–1.2 mm was ultimately adopted. The specific element counts, node counts, and mesh sizes for each model are detailed in Table 1.

**Table 1:** Mesh information of finite element models for different internal fixation methods in subtrochanteric femoral fractures

Model	Element Count	Node Count	Mesh Size
Long-nail combination	275,892	77,523	Adaptive mesh size of 1.0–1.2 mm
Medium-nail combination	315,795	86,996	Adaptive mesh size of 1.0–1.2 mm
Short-nail combination	215,597	61,936	Adaptive mesh size of 1.0–1.2 mm

The mesh components were saved as INP files and imported into Abaqus 6.14 finite element software (Dassault Systèmes, France) for preprocessing. Titanium alloy was treated as a continuous, isotropic, and linearly elastic material; accordingly, its elastic modulus was set to 110,000 MPa and its Poisson’s ratio to 0.3 [14]. Each bone element was assigned individual material values based on gray-value formulae. The gray-value method was applied to assign material properties to each element individually, with the apparent density (ρ) and elastic modulus (E) of each region derived from the CT-derived HU values according to the following formulae [15]:

1 $$\:\begin{array}{c}\rho\:\left(Kg/{m}^{3}\right)=131+1.067\times\:GV\left(\mathrm{H}\mathrm{U}\right)\end{array}$$

2 $$\:\begin{array}{c}E\left(MPa\right)=0.004\times\:{\rho\:}^{2.01}\left(Kg/{m}^{3}\right)\end{array}$$

To evaluate the influence of bone quality on construct biomechanics, each of the three internal fixation models was further divided into two subgroups: a healthy bone subgroup and an osteoporotic bone subgroup. In the healthy bone subgroup, bone material properties were assigned according to Eqs. (1) and (2) as described above. In the osteoporotic bone subgroup, the elastic modulus of each bone element was uniformly reduced to 60% of the corresponding healthy value, while Poisson’s ratio remained unchanged [16]. This simplified approach allows the mechanical consequences of reduced bone stiffness associated with osteoporosis to be captured within the existing CT-derived heterogeneous material framework.

#### Contact conditions, boundary conditions, and load settings

**Fig. 4:**
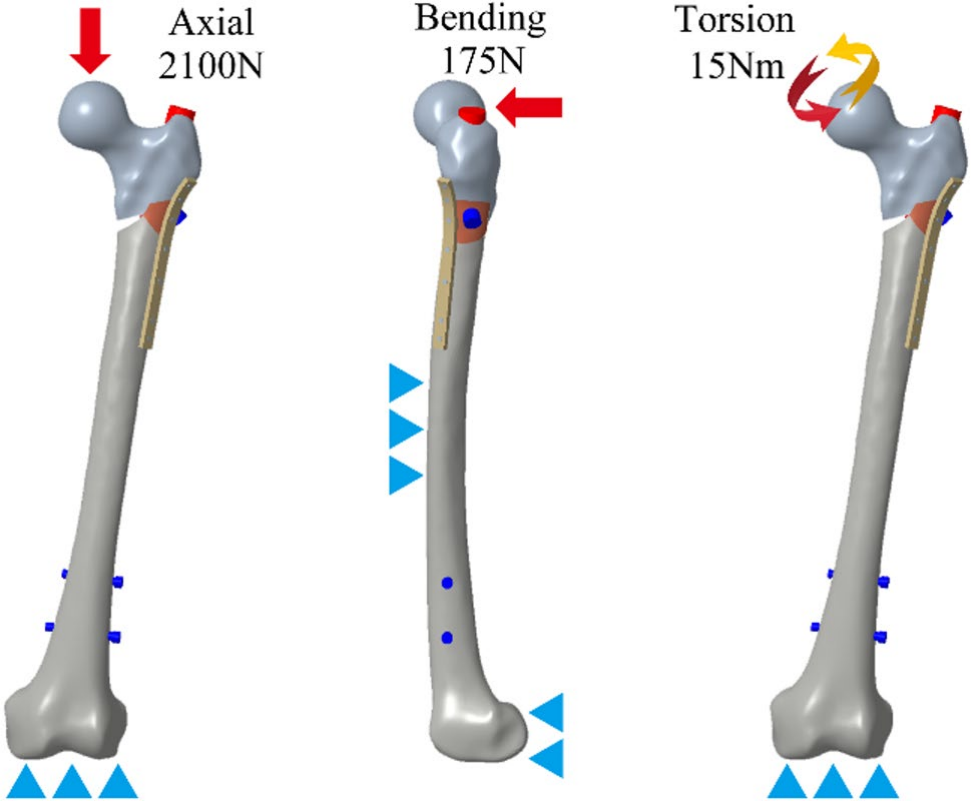
Loading and boundary conditions of the finite element model for subtrochanteric femoral fracture

All contact interfaces between components were defined as frictional contact. The coefficient of friction at bone–bone interfaces was set to 0.46, at bone–implant interfaces to 0.42, and between implant components to 0.20 [17]. Three biomechanically representative loading conditions were applied to each model to simulate the principal mechanical demands encountered during postoperative rehabilitation: (1) axial compressive loading, (2) lateral bending load, and (3) torsional loading. For the axial loading condition, a compressive load of 2,100 N—equivalent to approximately 300% of the volunteer’s body weight (70 kg)—was applied uniformly to the articular surface of the femoral head to replicate the peak hip-joint reaction force experienced during normal walking [18]. For the bending loading condition, a lateral force of 175 N was applied to simulate the bending moment generated at the proximal femur during activities involving lateral inclination [18]. For the torsional loading condition, a torque of 15 N·m was applied along the axis of the femoral neck to represent the maximum torsional load imposed on the femoral head during normal gait [18]. In all three loading conditions, the distal femoral condyles were fully constrained in all six degrees of freedom to prevent rigid body displacement during analysis (Fig. 4).

#### Modelling verification

To further support the validity of the present finite element framework, the displacement results obtained in this study were compared with those reported by Yang et al. [18], who applied identical loading conditions (axial 2,100 N, bending 175 N, torsion 15 N·m) to a finite element model of subtrochanteric femoral fractures and validated it experimentally against physical mechanical testing. Under axial loading, Yang et al. [18] reported a whole femoral displacement of 9.63 mm for PFNA fixation in Seinsheimer type IIIA fractures. In the present study, the medium-nail combination (PFNA-based, 240 mm) yielded a maximum femoral displacement of 10.7 mm under the same axial load in Seinsheimer type IV fractures. The modestly higher displacement observed in our model is consistent with the greater instability inherent to type IV fractures, which involve compromise of both the medial and lateral walls. This cross-study agreement in displacement magnitude under identical loading parameters and boundary conditions provides indirect support for the reliability of the present finite element models.

### Primary outcome measures

1. Overall displacement: The overall displacement of each nail-length combination model (long-nail, medium-nail, and short-nail combinations) was evaluated under axial, bending, and torsional loading conditions to assess construct stability.
2. Stress distribution: ① Femoral stress: The Von Mises stress distribution and magnitude across the femur were measured and analyzed for all three internal fixation models under each loading condition, with particular attention to stress concentration in the region adjacent to the fracture line and in the greater trochanter area. ② Intramedullary nail and reconstruction plate stress: The effect of intramedullary nail length on stress distribution within the internal fixation system was evaluated, with identification of stress concentration zones and assessment of implant failure risk.
3. Load-sharing ratio analysis: Load-sharing ratios were calculated based on the strain energy stored in each component (intramedullary nail, reconstruction plate, and femur) under each loading condition. The proportion borne by each component was expressed as its strain energy as a percentage of the total strain energy of the entire construct. The proportion of load borne by the intramedullary nail, reconstruction plate, and femur was analyzed under each loading condition to evaluate the effect of nail length on load transfer pathways. All stresses presented and analyzed in this study are Von Mises equivalent stresses, which integrate the principal stress components under a three-dimensional stress state and are commonly used to assess the risk of material yielding. The Von Mises stress (σₑ) is calculated using the following formula:

3 $$\sigma_e=\frac{1}{\sqrt{2}}\sqrt{\left(\sigma_1-\sigma_2\right)^2+\left(\sigma_2-\sigma_3\right)^2+\left(\sigma_3-\sigma_1\right)^2}$$

(σ₁, σ₂, and σ₃ denote the principal stresses in the first, second, and third directions, respectively).

## Results

### Comparison of overall displacement among models with different nail lengths

**Fig. 5:**
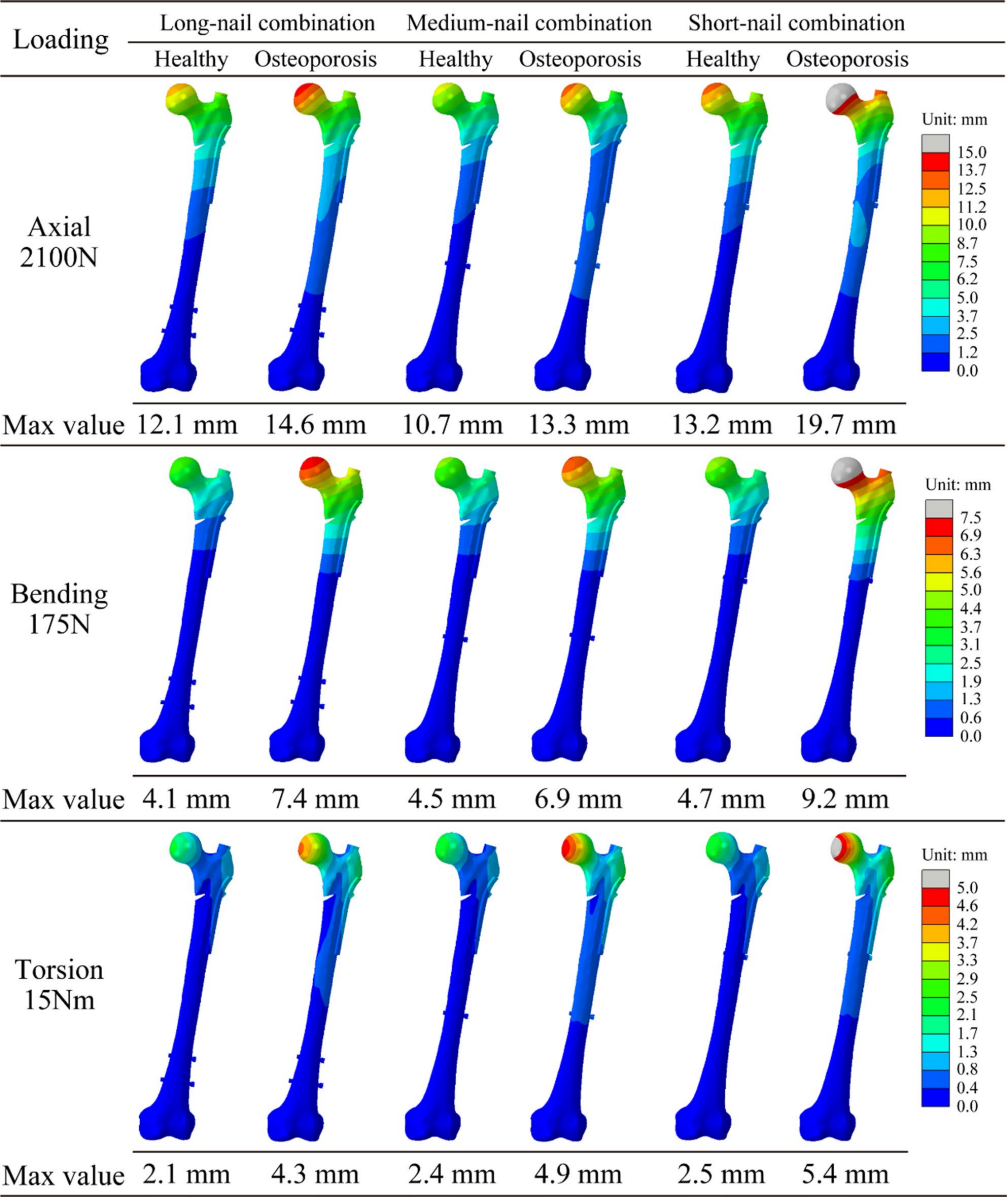
Overall displacement distribution contour maps of three models under different loading conditions

The maximum displacement values for all constructs under three loading conditions are summarised in Table 2, and the distribution contour maps are shown in Fig. 5. Under axial loading (2100 N), the medium-nail combination exhibited the smallest maximum femoral displacement in both the healthy (10.7 mm) and osteoporotic (13.3 mm) subgroups, while the short-nail combination produced the largest values (13.2 mm and 19.7 mm, respectively). The short-nail combination was also most sensitive to bone quality: its intramedullary nail displacement increased from 10.1 mm (healthy) to 18.5 mm (osteoporosis, + 83.2%), and reconstruction plate displacement rose from 4.7 mm to 7.8 mm (+ 66.0%), substantially exceeding the changes observed in the long-nail and medium-nail combinations. Under bending loading (175 N), the long-nail combination produced the smallest femoral displacement (4.1 mm in both subgroups), while the short-nail combination showed the largest and greatest bone-quality sensitivity (4.7 mm healthy vs. 9.2 mm osteoporosis; +95.7%). Under torsional loading (15 N·m), inter-group differences were minor across all nail lengths (range: 2.1–5.4 mm), though osteoporosis had more pronounced effects than in the axial and bending cases. Across all loading conditions, displacement contour maps showed a consistent proximal-to-distal gradient, with maximum values at the femoral head and near-zero values at the constrained distal condyles.

**Table 2:** Comparison of maximum displacements (mm) for the three models under different loading conditions

Parameter	Long-nail combination		Medium-nail combination		Short-nail combination	
	Healthy	Osteoporosis	Healthy	Osteoporosis	Healthy	Osteoporosis
Axial Load						
Intramedullary nail	11.5	13.8	10.1	12.4	10.1	18.5
Reconstruction plate	6.2	5.9	4.7	4.2	4.7	7.8
Femur	12.1	14.6	10.7	13.3	13.2	19.7
Bending Load						
Intramedullary nail	3.9	7.0	4.3	6.6	4.3	8.9
Reconstruction plate	1.6	3.6	1.9	3.2	1.9	4.5
Femur	4.1	7.4	4.5	6.9	4.7	9.2
Torsion Load						
Intramedullary nail	1.9	3.8	2.1	4.3	2.1	4.8
Reconstruction plate	1.2	2.1	1.2	2.2	1.2	2.6
Femur	2.1	4.3	2.4	4.9	2.5	5.4

### Comparison of femoral stress magnitude and distribution among models

**Fig. 6:**
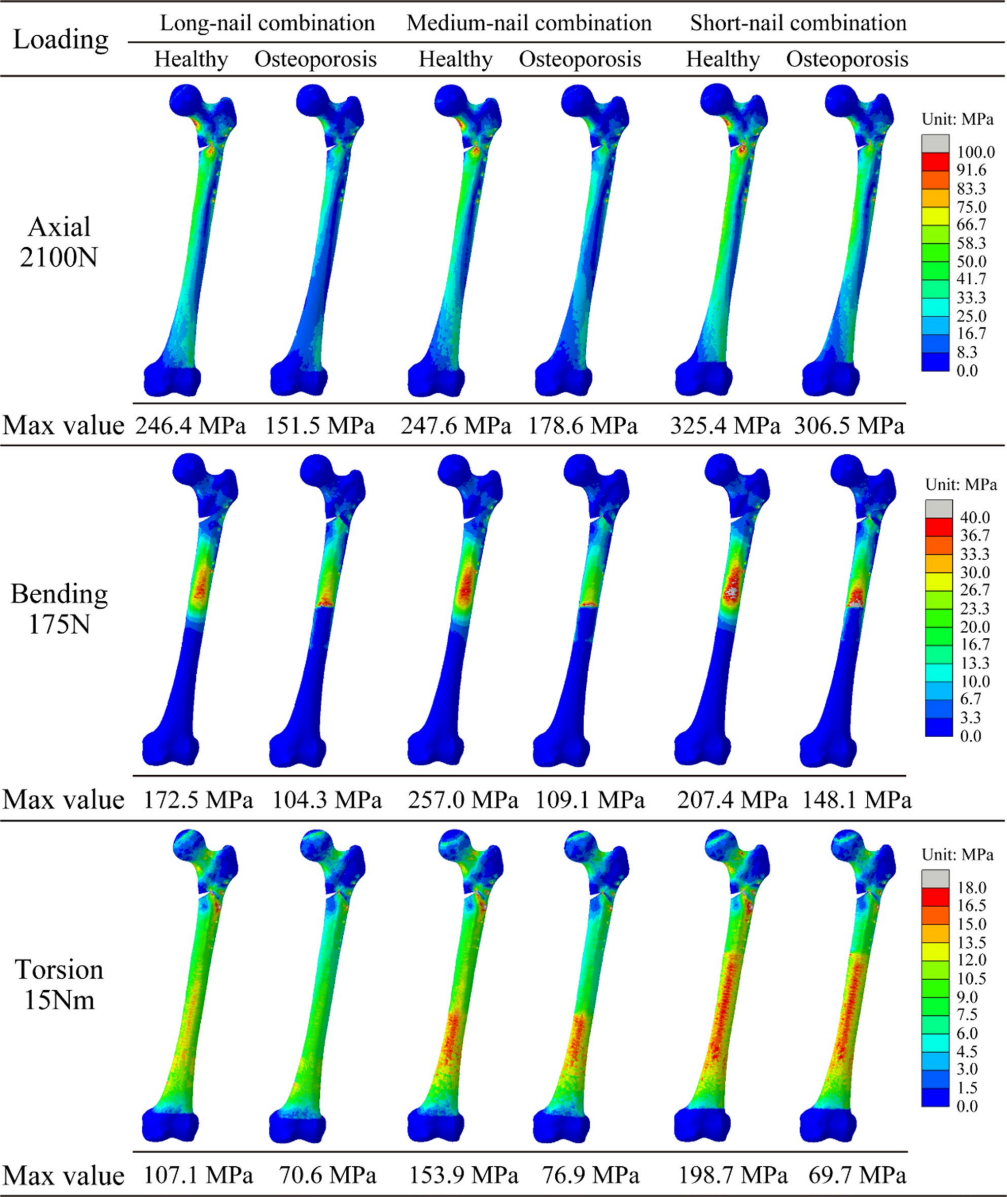
Von Mises stress distribution contour maps of femur in three models under different loading conditions. Note: Von Mises stress distribution of the femur

Von Mises femoral stress distributions are shown in Fig. 6 and summarised in Table 3. Under axial loading, the short-nail combination generated the highest maximum femoral stress (325.4 MPa healthy; 306.5 MPa osteoporosis), exceeding the long-nail combination by 32.1%; the medium-nail combination was intermediate (247.6 MPa; 178.6 MPa). High-stress zones were concentrated in the proximal medial cortex and greater trochanter region, with a notably larger high-stress area in the short-nail construct. Notably, the long-nail combination demonstrated substantial stress reduction under osteoporotic conditions (246.4 MPa healthy to 151.5 MPa osteoporosis, -38.5%), suggesting superior protective capacity through effective load transfer mechanisms. Under bending loading, the stress ranking differed from the axial case: the medium-nail combination yielded the highest femoral stress (257.0 MPa; 109.1 MPa), followed by the short-nail (207.4 MPa; 148.1 MPa) and the long-nail (172.5 MPa; 104.3 MPa) combinations. Under torsional loading, the short-nail combination again produced the highest femoral stress (198.7 MPa healthy), whereas the osteoporotic short-nail subgroup showed a reduction to 69.7 MPa, suggesting substantial load redistribution toward the implant under reduced bone stiffness. Across all conditions, osteoporosis caused variable effects on femoral stress (ranging from − 38.5% reduction in long-nail to -56% reduction in medium-nail under bending), confirming that the relative stress distribution among nail-length groups showed complex interactions with bone quality.

**Table 3:** Comparison of maximum stresses (MPa) for the three models under different loading conditions

Parameter	Long-nail combination		Medium-nail combination		Short-nail combination	
	Healthy	Osteoporosis	Healthy	Osteoporosis	Healthy	Osteoporosis
Axial Load						
Intramedullary nail	1001.0	1091.0	1045.0	1145.0	836.1	1043.0
Reconstruction plate	654.9	499.7	578.8	592.2	804.5	764.6
Femur	246.4	151.5	247.6	178.6	325.4	306.5
Bending Load						
Intramedullary nail	143.5	242.1	157.1	212.3	199.4	322.9
Reconstruction plate	168.9	219.0	203.7	173.8	190.4	208.6
Femur	172.5	104.3	257.0	109.1	207.4	148.1
Torsion Load						
Intramedullary nail	169.5	292.2	300.7	396.9	208.6	342.1
Reconstruction plate	152.1	154.4	186.6	201.4	182.6	176.3
Femur	107.1	70.6	153.9	76.9	198.7	69.7

### Comparison of intramedullary nail and reconstruction plate stress magnitude and distribution among models

**Fig. 7:**
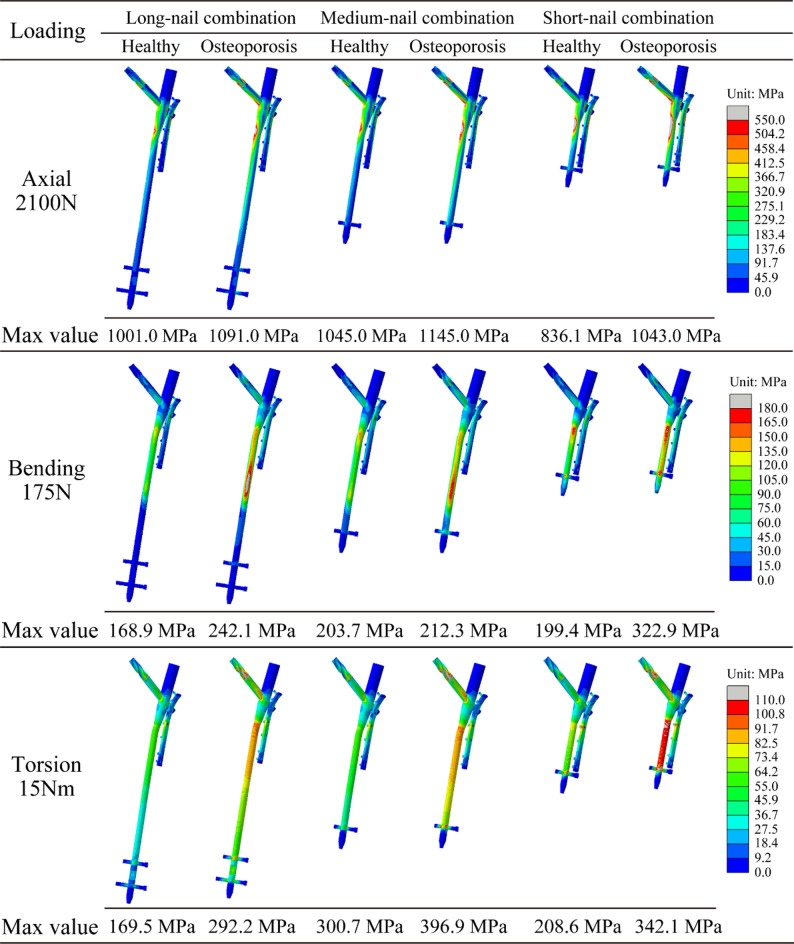
Von Mises stress distribution contour maps of intramedullary nail and reconstruction plate in three models under different loading conditions. Note: Von Mises stress distribution of the intramedullary nail and reconstruction plate

Implant stress contour maps are shown in Fig. 7, with maximum values presented in Table 3. Stress concentration was consistently located at the helical blade–main nail junction across all loading conditions. Under axial loading, the medium-nail combination produced the highest intramedullary nail stress (1045.0 MPa; 1145.0 MPa), followed by the long-nail (1001.0 MPa; 1091.0 MPa) and short-nail (836.1 MPa; 1043.0 MPa) combinations. The short-nail combination, despite lower nail stress under axial loading, compensated with markedly higher reconstruction plate stress (804.5 MPa), 22.9% and 39.0% greater than the long-nail and medium-nail combinations, respectively. Notably, the short-nail nail stress increased by 24.7% under osteoporotic conditions (836.1 to 1043.0 MPa), substantially exceeding the increases in the medium-nail (9.6%) and long-nail (9.0%) combinations, highlighting heightened vulnerability of the short-nail construct. Under bending loading, the short-nail combination generated the highest nail stress (199.4 MPa; 322.9 MPa), exceeding the long-nail combination by 33.4% in osteoporotic conditions. Under torsional loading, a distinct pattern emerged: the medium-nail combination produced by far the highest nail stress (300.7 MPa; 396.9 MPa), 77.4% above the long-nail (169.5 MPa) and 44.1% above the short-nail (208.6 MPa) combinations in healthy conditions, with further increases under osteoporotic conditions (396.9 MPa), suggesting a specific susceptibility of the medium-nail construct to torsional stress concentration. Osteoporosis had variable influence on implant stress across loading conditions (ranging from + 8.9% in long-nail under axial to + 62.0% in short-nail under bending), indicating that implant stress distribution is influenced by complex interactions between construct geometry and bone stiffness.

### Load-sharing ratio analysis

**Fig. 8:**
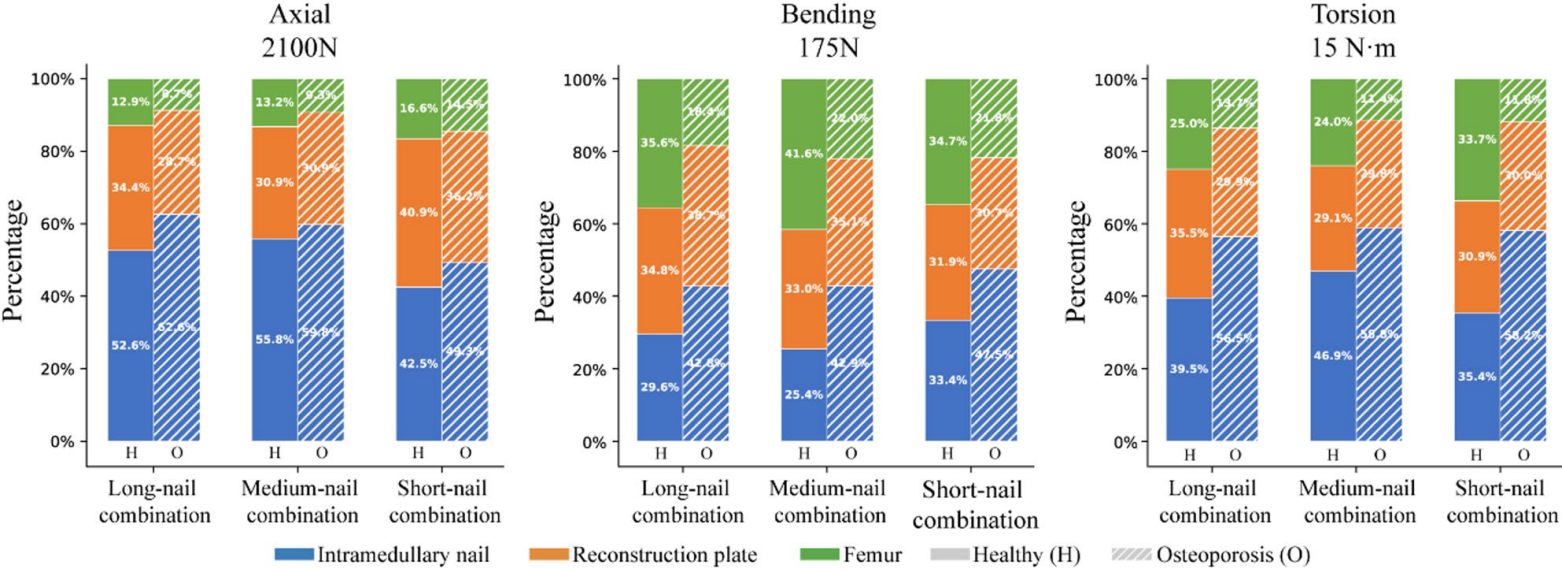
Load sharing ratio of each component in internal fixation system under different loading conditions

Load-sharing ratios under all three loading conditions are illustrated in Fig. 8. Across all constructs and loading modes, the intramedullary nail bore the largest load fraction (~ 47%-63%), followed by the reconstruction plate (~ 28%-41%), with the femur contributing the least (~ 5%-14%). The short-nail combination directed the highest proportion to the nail and the lowest to the femur under axial loading (49.3% and 14.5%, respectively), consistent with its shorter medullary support span. Notably, the long-nail combination demonstrated the most substantial shift in load distribution under osteoporotic conditions, with the intramedullary nail load increasing from 52.6% to 62.6% (+ 10.0% points), substantially exceeding the shifts in the medium-nail (+ 3.9% points) and short-nail (+ 6.8% points) combinations. In the osteoporotic subgroup, the femur bore approximately 3–5% points less load than in the healthy subgroup, with the difference redistributed to the two implants; the overall rank order among constructs was preserved across all conditions, though the magnitude of load redistribution varied significantly by construct type.

## Discussion

This study employed finite element analysis to compare the biomechanical performance of long-nail, medium-nail, and short-nail combinations—each paired with a reconstruction plate—for fixation of Seinsheimer type IV subtrochanteric femoral fractures under axial, bending, and torsional loading conditions in both healthy and osteoporotic bone subgroups. All three constructs provided effective mechanical support; however, the medium-nail combination demonstrated the best overall displacement control under axial loading, while the short-nail combination was associated with higher stress levels across multiple loading modes and greater mechanical vulnerability in osteoporotic bone.

Analysis of overall displacement showed that under axial loading, the medium-nail combination produced the smallest maximum femoral displacement (10.7 mm), lower than both the long-nail (12.1 mm) and short-nail (13.2 mm) combinations. This advantage can be explained from several perspectives. First, the overall stability of an intramedullary nail is closely related to the distal locking screw position; placing the screw at the mid-medullary canal—as in the medium-nail construct—optimises the bone–nail contact interface and enhances both rotational and axial stability [19, 20]. Second, an intermediate nail length improves load dispersal and reduces the lever-arm effect generated at the distal tip of excessively long implants, thereby enhancing overall stability [11, 21]. In the short-nail combination, the distal locking screw proximity to the fracture line shortens the load transfer pathway and forces the reconstruction plate—an eccentric fixation device with inferior bending resistance—to assume a disproportionate share of the load, undermining overall stability [9, 22]. Conversely, the excessively long moment arm of the long-nail combination generates additional bending moments at the distal femur that are detrimental to axial stability [23]. Under bending loading, the long-nail combination produced the smallest displacement (4.1 mm), suggesting that greater nail span provides superior resistance to lateral bending forces; this distinction is clinically relevant for patients who resume activities involving lateral inclination during early rehabilitation.

Stress analysis confirmed that stress concentration was consistently located at the helical blade–main nail junction across all loading conditions, consistent with prior studies [24–26]. Under axial loading, the short-nail combination generated the highest maximum femoral stress (325.4 MPa, exceeding the long-nail combination by 32.1%) and the highest reconstruction plate stress (804.5 MPa), attributable to its shorter intramedullary support span and less uniform load distribution. Furthermore, the high-stress zone adjacent to the fracture line was notably larger in the short-nail combination than in the other two constructs, indicating a greater risk of peri-implant bone failure under sustained loading. Under bending loading, the short-nail combination also produced the highest intramedullary nail stress (199.4 MPa, + 38.9% vs. the long-nail combination), further confirming that short-nail fixation imposes elevated mechanical demands on both the nail and plate across multiple loading modes. In contrast, the medium-nail and long-nail combinations maintained more uniform stress distributions, with high stress concentrated in the proximal helical blade region, which serves as the principal load-transfer node and thereby favours effective load dispersal through the central medullary fixation [27].

A notable and previously unreported finding of the present study was the markedly elevated intramedullary nail stress observed in the medium-nail combination under torsional loading (300.7 MPa), which exceeded the long-nail (169.5 MPa) and short-nail (208.6 MPa) combinations by 77.4% and 44.1%, respectively. This pattern differs substantially from the axial and bending loading results and warrants careful interpretation. Under torsional loading, load is transmitted primarily through the proximal helical blade and the bone–nail interface; at intermediate nail length, the geometric relationship between the distal locking screw position and the torsional neutral axis may create a more pronounced stress concentration at the helical blade junction than occurs with either shorter or longer constructs [11]. Although the torsional load magnitude employed (15 N·m) is representative of normal gait, this finding suggests that patients with medium-nail fixation who perform early rotational activities—such as pivoting or turning—may be subject to higher torsional nail stress than previously appreciated. Clinicians should therefore counsel all patients regarding rotational weight-bearing restrictions during the early postoperative period, regardless of nail length.

The osteoporotic bone subgroup revealed clinically relevant differences from the healthy subgroup. The short-nail combination showed the greatest sensitivity to bone quality deterioration: under axial loading, intramedullary nail displacement increased by 83.2% (10.1 to 18.5 mm) and reconstruction plate displacement by 66.0% (4.7 to 7.8 mm), substantially exceeding the changes observed in the long-nail and medium-nail combinations. This heightened displacement response likely reflects the combined effect of reduced bone stiffness and the already shortened load transfer pathway in the short-nail construct, where the reconstruction plate—an eccentric fixation device with inferior bending resistance—assumes a disproportionate share of the load, undermining overall stability [28]. In terms of femoral stress, the long-nail combination demonstrated the largest stress reduction under osteoporotic conditions (-38.5%), while the short-nail combination showed minimal reduction (-5.8%), indicating limited bone-protective capacity. Load-sharing analysis further showed that the femur bore approximately 3–5% points less load in the osteoporotic subgroup, with the long-nail combination demonstrating the most substantial load redistribution to the intramedullary nail (+ 10.0% points), suggesting an adaptive response that protects compromised bone; in contrast, the short-nail combination’s limited adaptive capacity may predispose it to accelerated fatigue damage accumulation. These findings indicate that for osteoporotic patients with Seinsheimer type IV subtrochanteric fractures, the short-nail combination carries a substantially higher risk of early fixation failure, and medium-nail or long-nail fixation combined with a reconstruction plate represents a more mechanically appropriate choice.

### Study strengths

The present study offers several strengths relative to existing literature. To our knowledge, this is among the first finite element studies to specifically examine nail length within a hybrid nail-plate construct for Seinsheimer type IV subtrochanteric fractures. Prior work by Je et al. [9], Gonzalo et al. [29], and Zhang et al. [30] addressed nail length in the context of standalone intramedullary nailing; none evaluated the hybrid configuration in which the reconstruction plate assumes a substantial share of eccentric load, fundamentally altering the biomechanical significance of nail length selection. Additionally, the concurrent inclusion of healthy and osteoporotic bone subgroups, three representative loading modes, and strain energy–based load-sharing analysis together provided a more comprehensive characterisation of construct mechanics than has been reported previously. Model plausibility was further supported by cross-study comparison with an experimentally validated finite element model under identical loading conditions [18]. The finding that a medium-length nail may offer superior axial stability over the longest available implant challenges the conventional assumption that “longer equals more stable,” and may be explained by the balance of load sharing among the nail, plate, and bone at intermediate length [31]: an excessively long nail creates distal stress concentration, while an excessively short nail results in excessive proximal load concentration, both of which are detrimental to overall stability.

### Study limitations and future directions

Several limitations should be noted. First, all models were derived from a single male volunteer, limiting generalisability across ages, sexes, and femoral morphologies; multi-subject or cadaveric validation is needed. Second, osteoporosis was approximated by a uniform 60% reduction in elastic modulus, which does not capture the anisotropic and heterogeneous nature of trabecular remodelling; future work incorporating patient-specific bone mineral density mapping would improve accuracy. Third, dynamic loading, muscle forces, and gait-cycle complexity were not simulated, and the frictional contact model does not reflect the evolving bone–implant interface during healing. Fourth, clinical validation is absent; prospective cohort studies with radiographic and functional follow-up are required to determine whether the biomechanical advantages of medium-nail fixation translate into improved outcomes such as reduced implant failure and earlier return to weight-bearing.

### Clinical implications

Based on the biomechanical findings of this study, the following clinical implications are proposed. First, for Seinsheimer type IV subtrochanteric femoral fractures, the hybrid fixation strategy combining a PFNA intramedullary nail with a reconstruction plate achieves effective load sharing across all physiologically relevant loading modes, providing a sound biomechanical rationale for its clinical application. Second, under axial loading—the dominant mechanical demand during weight-bearing rehabilitation—a medium-length intramedullary nail (240 mm) demonstrated potentially superior displacement control and may serve as the preferred option for the majority of patients with adequate bone quality. Third, although the short-nail combination offers operative advantages including a smaller incision and reduced intraoperative blood loss, it exhibits higher stress levels across multiple loading modes and markedly greater displacement sensitivity in osteoporotic bone; caution is therefore warranted in elderly osteoporotic patients or those requiring early weight-bearing, and an extended period of postoperative partial weight-bearing restriction should be considered for this subgroup. Fourth, the unexpected torsional stress concentration observed in the medium-nail construct (300.7 MPa, substantially exceeding both the long- and short-nail combinations) highlights the importance of advising all patients—regardless of nail length—to avoid early rotational loading activities such as pivoting and turning during the initial postoperative period. Fifth, the long-nail combination demonstrated the most pronounced adaptive load redistribution to the intramedullary nail under osteoporotic conditions (+ 10.0% points), suggesting a bone-protective mechanism that may be advantageous in patients with severely compromised bone mineral density. Sixth, clinical decision-making should therefore incorporate individualised assessment of fracture type, bone mineral density, femoral medullary canal morphology, and anticipated postoperative rehabilitation demands, rather than applying a uniform nail-length strategy across all patients.

## Conclusion

The medium-nail combination (240 mm) showed potentially favorable biomechanical stability for Seinsheimer type IV subtrochanteric femoral fractures when combined with a reconstruction plate, while the short-nail combination exhibited higher stress levels and greater mechanical vulnerability in osteoporotic bone. Intramedullary nail length should be individually selected based on fracture type, bone quality, and postoperative rehabilitation requirements.

## Data Availability

The datasets used and/or analyzed during the current study are available from the corresponding author upon reasonable request.

## Abbreviations

CT: Computed Tomography
DICOM: Digital Imaging and Communications in Medicine
FEA: Finite Element Analysis
HU: Hounsfield Units
IGS: Initial Graphics Exchange Specification
MPa: Megapascal
PFNA: Proximal Femoral Nail Antirotation
STL: Standard Tessellation Language
VMS: Von Mises Stress
